# Supplementary material for: Erratum
Source: Rev Bras Epidemiol. 2024 Sep 30;27:e220026erratum. doi: 10.1590/1980-549720220012.supl.1erratum (PMC11497708; doi:10.1590/1980-549720220012.supl.1erratum)
Supplement: Related texts

# Correction: Erratum

**DOI:** 10.1590/1980-549720220012.supl.1.1erratum

https://doi.org/10.1590/1980-549720220012.supl.1.1erratum

No artigo “**Tendência da incidência dos cinco principais tipos de câncer na Grande Cuiabá, 2000 a 2016**”, DOI: https://doi.org/10.1590/1980-549720220012.supl.1.1, publicado no periódico Rev Bras Epidemiol 2022; 25: e220012.supl.1:

**Na página 1 foi incluído:**

EDITORAS ASSOCIADAS: Elisete Duarte http://orcid.org/0000-0002-0501-0190, Gulnar Azevedo e Silva http://orcid.org/0000-0001-8734-2799

EDITORA CIENTÍFICA: Cassia Maria Buchalla http://orcid.org/0000-0001-5169-5533
